# Advancing Palliative Care Systematically Using Education Through a Grassroots Coalition

**DOI:** 10.1089/pmr.2022.0055

**Published:** 2023-03-22

**Authors:** Sheri Kittelson, Margaret C. Lo, Melanie Hagen, Paige C. Barker, Raed Al Yacoub, Paulette Hahn, Paula Turpening, Diana J. Wilkie, Celine Cattier, Tracy Romanello, Sloan B. Karver, Belina Adkins-Dodd, Adriana S. Shum-Jimenez, Neal Weisbrod, Paul A. Ledford, Susan Ponder-Stansel

**Affiliations:** ^1^ Department of Medicine, University of Florida, Gainesville, Florida, USA.; ^2^ College of Nursing, University of Florida Health, Gainesville, Florida, USA.; ^3^ Catholic Hospice and Catholic Palliative Care Services, Miami Lakes, Florida, USA.; ^4^ United Healthcare Community Plan, Hot Springs, Arkansas, USA.; ^5^ Community Hospice and Palliative Care, Jacksonville, Florida, USA.; ^6^ Florida Hospice and Palliative Care Association, Tallahassee, Florida, USA.; ^7^ Alivia Care, Inc., Jacksonville, Florida, USA.

*Dear Editor:*

The needs of an aging population and advancements in the treatment of both chronic and life-limiting illness have increased demand for quality palliative care (PC). Despite being home to one of the nation's largest elderly populations, the state of Florida had no formal program or comprehensive plan to standardize PC. In response, a steering group from >30 organizations formed the Florida Palliative Care Coalition (FPCC). Its mission is to “promote better quality of life for those living with serious, chronic, or advanced illness through research, education, advocacy, and access to holistic health care.”^1^

The organization was formed in 2018 and now serves as the only organization solely focused on advancing the understanding, visibility, availability, and practice of PC in Florida. The coalition vision is a future in which Floridians with serious illness and their caregivers receive holistic health care that aligns with their goals, values, and preferences and helps improve quality of life.

Health care providers of the future will need to be well prepared to provide expert symptom management and address the holistic needs (physical, psychosocial, and spiritual) of patients dealing with serious illness. Such preparation begins with medical education. The FPCC hosted its first summit in Summer 2021 bringing together champions from multiple organizations around the state. The summit held an educational session for facilitators to solicit ideas on the ideal state of palliative education to include undergraduate medical education, graduate medical education, and continuing medical education (CME).

After the summit, volunteer members joined an education and practice work group to bring these ideas forward using quality improvement specific, measurable, achievable, relevant, timely (SMART) goals. The multidisciplinary team from various organizations met quarterly. The aim was to refine the summit ideas into two high-impact deliverable educational goals by 2022.

The work group defined SMART goal no. 1 to offer free PC education through the Florida Medical Association (FMA). Founded in 1874, the FMA represents >25,000 allopathic and osteopathic physicians on issues of legislation and regulatory affairs, medical economics and education, public health, and ethical and legal issues. The FMA offers education on communication tools for difficult conversation and education on conducting a family meeting or goals-of-care conversation.

As of 2022, FMA did not offer other specific PC education. In response, the FPCC education work group created a free case-based PC educational video in 2022 hosted on the University of Florida CME website covering PC for the dementia patient.^2^ Information on this free CME was sent in the January 5^th^, 2023, newsletter to FMA members.^3^

The long-term goal of the FPCC educational work group is to standardize PC education across Florida medical schools. SMART goal no. 2 aims to (1) systematically assess the current state of medical student education through review of national medical school curriculum, (2) review the literature on the current PC education landscape, and (3) survey Florida medical schools to establish current PC practices. Through this goal, FPCC discovered that currently the Liaison Committee on Medical Education (LCME) only recommends end-of-life and not PC education.

Yet, the 2017–2018 LCME Annual Medical School Questionnaire reported that 86% of the 147 participating medical education programs included PC in “preclerkship,” and 93% of the 147 programs reported included PC in their “clerkship” curriculum as either a required course or an elective course (Figure 1).^4^ A comprehensive literature review of PC education found consistency in the content being delivered within medical schools. The most frequently taught topics include attitudes toward death and dying, communication skills, and pain management. Pediatric and religious/cultural issues are less frequently taught.^5^

**FIG. 1.**
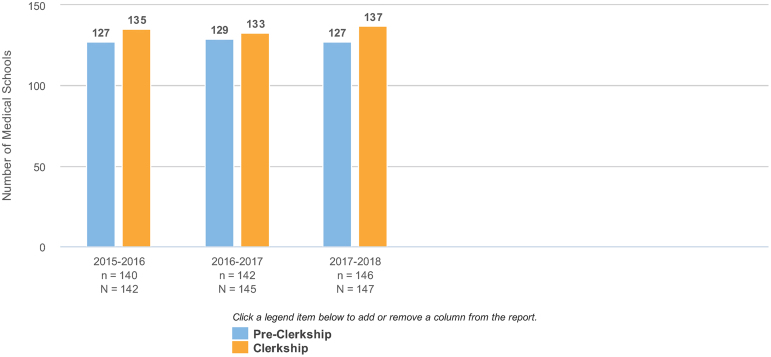
Number of medical schools including topic in required courses and elective courses: PC. PC, palliative care.

Finally, to further understand the current landscape of PC education, the coalition conducted a qualitative survey of Florida medical schools. The survey focused on the following domains: presence or absence of education, elective or required, didactic and/or clinical, and topics taught. The survey response rate was 38% (three out of eight schools). Of the responders, 100% reported a formal PC educational curriculum including 66% of schools with a required rotation and 33% with an elective rotation of one-week duration. In addition, 100% had didactic lectures on the following topics: pain and symptom management, communication (delivery of bad news), advance care planning, capacity assessment, and ethics and law related to end-of-life care.

The FPCC plans to meet again in 2023 and establish further goals based on the mentioned findings. The advances in PC medical education are pleasantly surprising given the lack of LCME-required medical student education. Discussion for next steps includes applying similar efforts to other relevant disciplines including social work. On a national level, the FPCC continues to support efforts from the American Academy of Hospice and Palliative Care's Palliative Care and Hospice Education and Training Act (PCHETA) to fund educational centers and workforce development.^6^ Advocacy for education on the national level is imperative to systematically address and provide standardized evidence-based PC curricula in medical education taught by a competent and adequate workforce.

## Abbreviations Used

CME: continuing medical education
FMA: Florida Medical Association
FPCC: Florida Palliative Care Coalition
LCME: Liaison Committee on Medical Education
PC: palliative care
PCHETA: Palliative Care and Hospice Education and Training Act
